# Platelets Retain High Levels of Active Plasminogen Activator Inhibitor 1

**DOI:** 10.1371/journal.pone.0026762

**Published:** 2011-11-01

**Authors:** Helén Brogren, Karin Wallmark, Johanna Deinum, Lena Karlsson, Sverker Jern

**Affiliations:** 1 Wallenberg Laboratory for Cardiovascular Research, Institute of Medicine, University of Gothenburg, Gothenburg, Sweden; 2 Bioscience, AstraZeneca R&D Mölndal, Mölndal, Sweden; Leiden University Medical Center, Netherlands

## Abstract

The vascular fibrinolytic system is crucial for spontaneous lysis of blood clots. Plasminogen activator inhibitor 1 (PAI-1), the principal inhibitor of the key fibrinolytic enzyme tissue-type plasminogen activator (tPA), is present in platelets at high concentrations. However, the majority of PAI-1 stored in platelets has been considered to be inactive. Our recent finding (Brogren H, *et al*. Blood 2004) that PAI-1 *de novo* synthesized in platelets remained active for over 24 h, suggested that PAI-1 stored in the α-granules might be active to a larger extent than previously reported. To re-evaluate this issue, we performed experiments where the fraction of active PAI-1 was estimated by analyzing the tPA-PAI-1 complex formation. In these experiments platelets were lysed with Triton X-100 in the presence of serial dilutions of tPA and subsequently the tPA-PAI-1 complex was evaluated by Western blot. Also, using a non-immunologic assay, tPA was labeled with ^125^I, and ^125^I-tPA and ^125^I-tPA-PAI-1 was quantified by scintigraphy. Interestingly, both methods demonstrated that the majority (>50%) of platelet PAI-1 is active. Further analyses suggested that pre-analytical procedures used in previous studies (sonication or freezing/thawing) may have substantially reduced the activity of platelet PAI-1, which has lead to an underestimation of the proportion of active PAI-1. Our *in vitro* results are more compatible with the role of PAI-1 in clot stabilization as demonstrated in physiological and pathophysiological studies.

## Introduction

*In vivo* clot lysis results primarily from activation of the fibrinolytic system by tissue-type plasminogen activator (tPA) released from the vascular endothelium. The thrombolytic activity of tPA is regulated by specific inhibitors, the most important of which is plasminogen activator inhibitor 1 (PAI-1). Blood clots contain large amounts of PAI-1 that may originate from α-granules of activated platelets [1]. Immuno-histochemical studies have shown that platelet-rich arterial clots contain 2 to 3 fold more PAI-1 than venous clots [2], [3], and there is a close correlation between the relative PAI-1 content of a clot and its resistance to thrombolysis [4]. The importance of platelet PAI-1 is further supported by *in vitro* clot assays on platelets from a patient with complete lack of PAI-1 expression [5], as well as by studies on thrombi generated in the Chandler loop experimental thrombosis model [6], [7]. Furthermore, studies in transgenic mice have shown that PAI-1 not only influences the resistance to thrombolysis but also the rate of progression of thrombus formation following vascular injury [8].

These observations, that clearly indicate an important physiological function of platelet PAI-1, have been difficult to reconcile with the fact that most previous studies have shown that only 2% to 5% of PAI-1 in platelets is active *e.g.* [9], [10], [11], [12]. Therefore, the role of platelet PAI-1 for clot stabilization has remained enigmatic. Following a recent study of the *de novo* synthesis of PAI-1 in platelets [13], we unexpectedly found that in a functional assay in which platelets were lysed in the presence of tPA, not only the small fraction of newly synthesized PAI-1, but also the majority of PAI-1 already present in the platelet apparently was able to complex-bind tPA. This observation suggested that the main proportion of platelet PAI-1 was active, but that pre-analytical conditions and/or the timing of the addition of tPA might be critical for correct assessment of the true PAI-1 activity.

In the studies cited above, platelets were lysed by ultrasound sonication [9], [10], [12]. However, it has been demonstrated that sonication *per se* may denature proteins and cause epitopes to be destroyed or hidden due to aggregation [14]. Thus, it might be possible that sonication used in the preparation of platelet lysates may induce latency transition, or protein damage that reduces the activity of PAI-1. Other commonly used platelet lysis protocols *e.g.* freezing/thawing or use of Triton X-100 can also accelerate inactivation of PAI-1 [15], [16]. Unless tPA is present already during lysis of the platelets, it might be possible that these procedures have lead to an underestimation of PAI-1 activity or, at least, caused a great variability depending on how much the inactivation rate is affected. Indeed, in a study of Wiman and co-workers on Triton X-100 lysed platelets, substantially higher PAI-1 activity levels were found with a wide inter-individual variability [17], [18].

In the present work we reinvestigated the issue of the activity of PAI-1 stored in washed platelet using a functional approach, studying the tPA-PAI-1 complex formation with two methods. Due to the conformational changes in the PAI-1 molecule depending on its state, detection and quantification using antibodies is very intricate. To avoid the difficulties of immunochemical detection of the diverse PAI-1 molecule, detection of tPA, either free or in complex with PAI-1, was used to determine the amount of active PAI-1. We also investigated the effect of different lysis methods on PAI-1 activity. The results show that the majority of platelet PAI-1 (>50%) is active and that the previous observations of low PAI-1 activity may be underestimations due to inactivation during the pre-analytical procedures.

## Results

### Total PAI-1 antigen in washed platelets

ELISA was used to determine the total amount of PAI-1 antigen in platelets, and the mean PAI-1 concentration was 0.79 (±0.13) ng/10^6^ platelets. Initially we used three different commercially available ELISA kits and the total mean of all three assays was 0.64 (±0.04) ng/10^6^ platelets. However, here we choose to report the results from the kit with the highest antigen concentrations to circumvent an overestimation of the level of activity.

### PAI-1 activity in lysed washed platelets determined by Western blot

**Figure 1:**
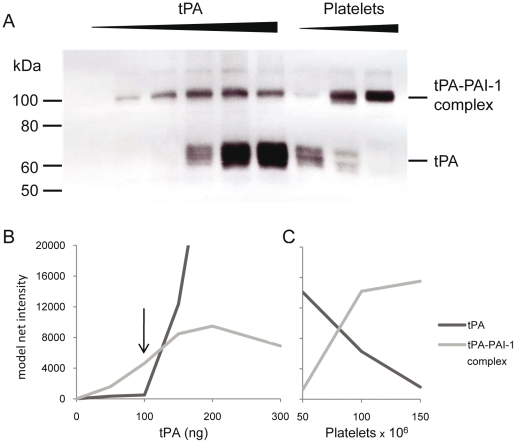
Western blot analysis of tPA-PAI-1 complex in dilutions of platelets and tPA. A. The platelets were incubated with tPA and were lysed in buffer with Triton X-100. Lane 1–6; constant number of platelets (in this case 400×10^6^) lysed in the presence of increasing amounts of tPA (0 ng–300 ng). Lane 7–9; constant amount of tPA (100 ng) and increasing number of platelets (50×10^6^–150×10^6^). The membrane is incubated with tPA mab PAM-3. B and C. Densitometric evaluations of the tPA and the tPA-PAI-1 complex. In fig B the band intensity is plotted against the tPA concentration in the sample. In figure C the intensity is plotted against the number of platelets (×10^6^) in the sample. The arrow indicates the tPA concentration selected for PAI-1 activity calculation.

Western blot analysis of platelet lysates was performed with both a tPA and a PAI-1 specific monoclonal antibody (mab). As shown in Figure 1, the amount of the tPA-PAI-1 complex increased with increasing tPA concentrations until a molar excess of tPA was reached. When the amount of tPA exceeded the amount of active PAI-1, a 68 kDa band appeared, representing free tPA. The highest molar concentration of tPA added without detection of free tPA was used to calculate the molar concentration of active PAI-1, assuming a 1∶1 stoichiometric complex and a molecular weight of 47 kDa and 68 kDa for PAI-1 and tPA, respectively. The increase of the tPA-PAI-1 complex was verified using the PAI-1 mab and the same dose-dependent response of the complex was observed (data not shown).

In order to separate tPA and PAI-1 properly, SDS has to be present in the electrophoresis. Treatment with SDS before separation can potentially cause dissociation of the complex and/or activation of latent PAI-1. However presence of SDS during the separation is not expected to affect the molecular forms of PAI-1 [19]. In this study there was no detectable difference between samples prepared with or without SDS in the sample buffer.

The results of the western blot in Figure 1A are also shown as densitometric evaluations of tPA and the tPA-PAI-1 complex in Figure 1 B and C. To clarify the course of action the details of this specific experiment are as follows: Figure 1A (left) and 1B; platelet count was constant in all lanes (400×10^6^), tPA was titrated by 50 ng per lane, ranging from 0–300 ng. Figure 1A (right) and 1C; tPA was constant in all lanes (100 ng), platelets were titrated by 50×10^6^ per lane ranging from 50–150×10^6^ platelets. The arrow indicates the tPA concentration used for calculating the amount of active PAI-1. The PAI-1 antigen concentration in these specific samples was 0.67 ng/10^6^ platelets by Coaliza (0.45 ng/10^6^ by TintElize and 0.42 ng/10^6^ by Immubind). The amount of active PAI-1 in this example is 145 ng which is equimolar to 100 ng tPA (arrow). Compared to the antigen level in 400×10^6^ platelets the amount of active PAI-1 is 54% using Coaliza (81% using TintElize and 86% using Immubind).

The average amount of functionally active PAI-1 from the 12 donors was 65±5.0%. Using the average PAI-1 concentration from the three different ELISAs (0.79±0.13, 0.48±0.08, and 0.64±0.23 ng/10^6^ for Coaliza, TintElize and Immubind respectively) the fraction of functionally active PAI-1 was 81 (±9)%.

### Activity of PAI-1 released from activated platelets studied by Western blot

**Figure 2:**
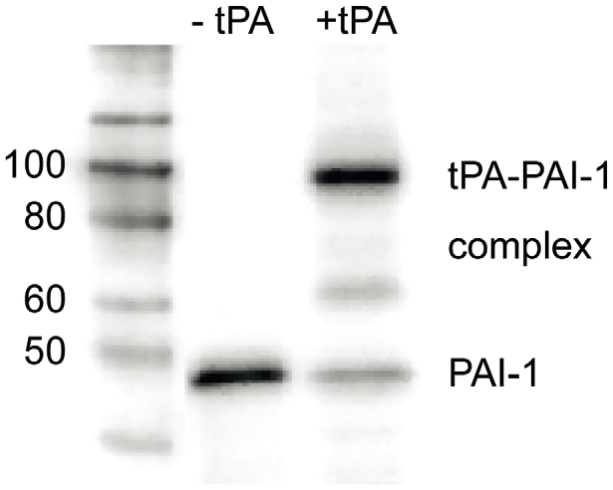
Western blot analysis of releasate from platelets activated by SFLLRN. Platelets (200×10^6^) activated by 25 µM SFLLRN in the absence or presence of an excess of tPA (1 µg). PAI-1 and tPA-PAI-1 complex detected by the PAI-1 mab, MAI-12.

In order to study the activity of PAI-1 released from activated platelets, PAI-1 and the tPA-PAI-1 complex were detected by Western blot from platelets activated by SFLLRN in the absence or presence of a molar excess of tPA. The majority of PAI-1 appears to complex bind tPA as shown in Figure 2. It is important to note that it is not possible to draw any conclusions of the activity of PAI-1 from the densitometric differences between the samples. This is due to the different affinity of the antibody (MAI-12) for the different forms of PAI-1. Nineteen different antibodies were tested for their affinity for active, latent, and complex bound PAI-1 but none of them had the same affinity for the different forms, as shown [20]. Therefore, it was not possible to use densitometric evaluation of PAI-1 western blots to quantify the proportion of active PAI-1.

### PAI-1 activity determined using ^125^I-tPA

**Figure 3:**
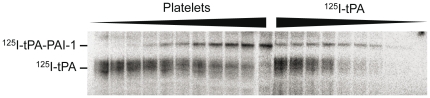
Autoradiography of ^125^I-tPA-PAI-1 complex in dilutions of platelets and ^125^I-tPA. Lane 1–11: increasing number of added platelets, from 20×10^6^ and 500×10^6^, lysed in the presence of 100 ng tPA. Lane 12–20: 250×10^6^ platelets lysed in presence of decreasing amounts of tPA (400 ng–25 ng).

To verify the results of the Western blot analysis, we performed a non-immunochemical assay using ^125^I-labelled tPA. The use of ^125^I-tPA and quantification by scintigraphy made it possible to determine the amount of ^125^I-tPA added without further increase of the ^125^I-tPA-PAI-1 complex (Figure 3). The highest concentration of ^125^I-tPA added without reaching maximum binding was compared to total PAI-1 in the samples determined by ELISA as described above. The mean PAI-1 activity in the three samples was 53±3.2%.

Using the average PAI-1 concentrations from the three ELISAs the activity was 72±6,1% (53±3.2, 82±5.6, and 82±13.4% for Coaliza, TintElize, and Immubind respectively). Thus, the experiments with ^125^I labelled tPA confirmed the results from the Western blot analysis with activities over 50%.

### Activity of PAI-1 in lysates from sonicated or freezed/thawed platelets

**Figure 4:**
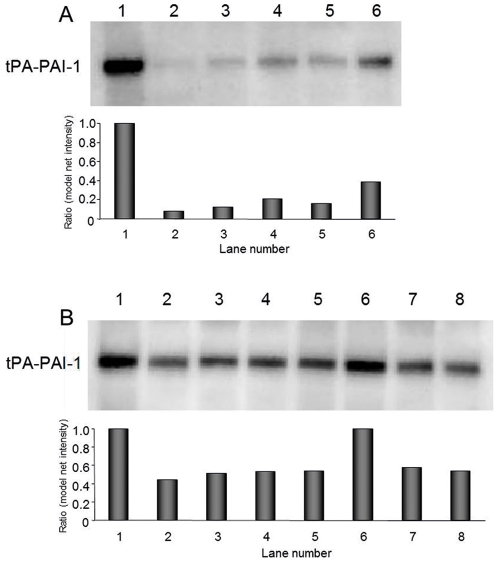
Western blot analysis of tPA-PAI-1 complex showing the differences in PAI-1 activity depending on the lysis method. In all samples, equal number of platelets (180×10^6^) was lysed and equal amounts of tPA (500 ng) was used. The formation of tPA-PAI-1 complex was detected by the PAI-1 mab (MAI-12). Each lane represents different lysis conditions. Sonicated samples on membrane A was lysed with high energy on setting 7. On membrane B, sonicated samples were lysed with milder sonication on setting 2. To lane 1, 5, 6, and 7 tPA was present during lysis. In lane 2, 3, and 4 tPA was added to the samples after lysis. Lysis conditions of the platelets: Lane 1A, 1B: in lysis buffer containing 0.1% Triton X-100 with tPA present. Lane 2A, 2B: in homogenisation buffer lysed by sonication. Lane 3A, 3B: in Pipes buffer lysed by sonication. Lane 4A, 4B: in Pipes buffer lysed by freezing and thawing. Lane 5A, 5B: in Pipes buffer lysed by sonication with tPA present. Lane 6A: in Pipes lysed by freezing and thawing with tPA present. Lane 6B 7B and 8B: 6 same as sample 1, 7 and 8 same as 3 and 4, but with 0.1% Triton X-100 added after sonication or freezing/thawing. The results of the densitometry measurements are presented as arbitrary units with samples lysed by Triton X-100 set to 1.0.

To assess whether the method of platelet disruption might affect the PAI-1 activity the results from lysis with Triton X-100 in the presence of tPA were compared with a series of experiments using sonication and freezing/thawing according to the previously used protocols. Sonication dramatically reduced the activity as shown in Figure 4. Furthermore, there appeared to be a dose-response relationship between the amount of sonication energy and degree of inactivation, since the activity in samples sonicated with high energy was considerably lower (Figure 4A) compared to samples prepared with low energy (Figure 4B). Analysing the ratio between densitometric intensity (model net intensity) of the tPA-PAI-1 bands, sonication with high energy reduced the activity by approximately 90% whereas with low energy there was a 50–60% reduction.

There was no significant difference in complex formation between samples sonicated with or without tPA present, indicating that sonication alone caused the reduction in activity and that it was not merely an effect of freezing/thawing of the samples after sonication. The reduction in activity was similar between samples sonicated and lysed by freezing/thawing. However, samples freeze/thawed with tPA present had higher activity than samples in which tPA was added after lysis. Addition of 0.1% Triton X-100 to the sonicated and freeze/thawed samples did not affect the results of the Western blot. We were unable to determine the effect of sonication of platelets in plasma due to too high protein content for ideal separation with SDS-PAGE (data not shown).

The effect of sonication of reactivated PAI-1 from the control plasma from Biopool was determined by Chromolize activity assay. The activity of the control plasma was 33.9 IU/ml. There was no significant difference between samples sonicated 5×5 s and 10×5 s (16.1 and 18.6 IU/ml with high energy and 29.4 and 33.6 IU/ml with low energy respectively). By average, sonication with high energy reduced the activity 49±3.3% and with low energy 7±4.0%.

## Discussion

In the present study we reinvestigated the important issue of the activity of platelet PAI-1 with a simple and direct functional approach in which the reaction between tPA and PAI-1 was studied by two assays based on reciprocating serial dilutions of tPA and platelets. Total PAI-1 antigen was determined using commercial ELISA kits, and tPA and tPA-PAI-1 complex was studied by Western blot analysis as well as with autoradiography and scintigraphy of ^125^I-labelled tPA. The study shows that the activity of platelet PAI-1 is considerably higher than previously reported in most studies. The average PAI-1 activity was estimated to 65% in samples analysed by Western blot and 53% in samples analysed with ^125^I-labelled tPA. Our results show that both sonication and freezing/thawing of the samples substantially reduced the detected PAI-1 activity, which may explain the low activity observed in studies using these lysis protocols.

Platelets contain large amounts of PAI-1 and the major part (approximately 90%) of blood PAI-1 is found in the platelet compartment. According to the traditional view, platelet PAI-1 is synthesized during the megakaryocyte stage, but we have shown that there is an on-going *de novo* synthesis of PAI-1 also in platelets [13]. Regardless of tissue origin, PAI-1 is synthesized in an active configuration but spontaneously converts to a thermodynamically more stable inactive form. The half-life of active PAI-1 is approximately 1–2 h at 37°C and pH 7.4 [21], [22], and only the active form of PAI-1 is capable of forming complex with, and irreversibly inhibit tPA [23]. It has generally been assumed that there is a similar rapid spontaneous inactivation of PAI-1 in the megakaryocyte and platelet, which might explain the low activity of platelet PAI-1 observed in most studies [9], [10], [11], [12].

However, both our own data and those of other investigators have suggested that platelets may possess a mechanism to preserve PAI-1 in the active configuration for longer periods of time [12], [13]. To investigate this hypothesis, it is critical that the method used to isolate PAI-1 from the platelet is able to capture the molecule in its active form and that spontaneous inactivation during the preparatory procedure is prevented. Conventional enzymatic assays for PAI-1 activity are inappropriate for this purpose and multicenter evaluations have shown that the majority of assays fail to correctly determine the true activity of prepared samples [24], [25], a conclusion that was confirmed by inconsistent and disparate results in our pilot studies (data not shown).

In agreement with our findings Fay et al [26] showed that the amount of active PAI-1 in a porcine coronary artery thrombi was 36%–50%. However, this result could not be confirmed in *in vitro* activated human platelets, although gentle conditions for PAI-1 isolation were used. One reason for this might be that neither tPA was present at the time of platelet activation, nor were any other actions taken to stabilize the active form of PAI-1 which could therefore spontaneously have been inactivated during the long time of extraction. To ensure an immediate capture of active PAI-1 at the time of lysis and to circumvent the limitations of enzymatic methods, we used an approach in which tPA was present already when the washed platelets were lysed. By subsequent direct detection of tPA and tPA-PAI-1 complex formation with antibodies and ^125^I-tPA, the intricate interactions of the platelet lysate with the enzymatic assays are avoided.

Both detection methods indicated that at least 50–70% of PAI-1 in washed platelets was present in an active configuration that was biologically functional and could bind tPA. Using a conservative definition of the amount of active PAI-1 by using the tPA concentration immediately below the maximum of complex formation, our approach may even have lead to an underestimation of the true amount of active PAI-1. Also, calculation of the proportion of active PAI-1 is dependent on the PAI-1 antigen assay used. In this study PAI-1 antigen was determined by three different ELISA assays which detect all molecular forms of PAI-1 with similar efficiency [11], [20], [27], [28]. We report the activity concentrations calculated from the assay that measured the highest antigen concentrations (Coaliza) to avoid a possible overestimation of the activity level. The ELISA assays are optimised for plasma samples, but the concentration of platelet PAI-1 is in accordance with previous reported levels [9], [11], [17] and variations between the assays are probably due to inter-assay variations previously described [29].

A limitation of the functional assay approach is that it only gives an approximate estimate of the activity, since it is limited by the tPA titration intervals. By decreasing the intervals, a 10% difference in the concentration of active PAI-1 could be detected.

To shed light on possible mechanisms behind the low activity rates observed in previous studies [9], [10], [11], [12], we investigated the influence of commonly used pre-analytic procedures. First, we studied the effect of sonication, since a recent study has demonstrated that energy levels as low as 30 W may cause protein damage [14] and it is conceivable that a thermodynamically unstable molecule, such as active PAI-1, is more susceptible to inactivation. Indeed, our results showed that even reactivated plasma PAI-1, stabilized by low pH, is very sensitive to sonication; its activity was reduced by approximately 50% with an energy load of 30 W, which is 5-fold lower than the energy used for platelet lysis [9]. Using the high-energy sonication protocol employed in previous reported studies, we found that platelet PAI-1 activity was reduced approximately 90%. Taken together, with the activity rates observed in the present study, one would expect sonication to reduce platelet PAI-1 activity to 7–8%, i.e. to similar levels as reported in previous studies.

The magnitude of the reduction in PAI-1 activity was similar when freezing/thawing was used for platelet lysis. However, whereas the reduced activity by sonication was independent of whether tPA was added before or after lysis, the underestimation of activity by freezing/thawing could partially be prevented by adding tPA before lysis. Another common procedure for platelet disruption is to use detergents such as Triton X-100 [17], [30]. However, it has been shown that Triton X-100 decreases the half-life of active PAI-1 markedly, and 0.2% Triton X-100 decrease the functional half-life of PAI-1 to less than 1 minute at 37°C [15], [16]. Therefore, also with such protocols it is crucial to add tPA before lysis. Since addition of Triton X-100 is not physiological and may facilitate the binding of tPA and PAI-1, we investigated if Triton X-100 affected the results of the Western blot analysis. However, when Triton X-100 was added to the platelets lysed by sonication and freezing/thawing no such enhancement was observed (figure 4B).

A potential concern in the present study could have been that the procedure we used in some way could have reactivated PAI-1 although it was in fact inactive *in vivo*. *In vitro* PAI-1 can be reactivated by denaturants such as SDS, guanidine HCl, and urea [31], and it has also been suggested that negatively charged phospholipids exposed on the surface of activated platelets could reactivate PAI-1 [32]. On the other hand, it has been reported that SDS may cause dissociation of the tPA-PAI-1 complex [19]. To rule out the possibility that our results were due to reactivation and/or dissociation of the tPA-PAI-1 complex formed, we performed a series of experiments both with and without SDS in the loading buffer before electrophoresis. However, these studies showed no detectable differences in PAI-1 activity whether SDS was present or not. This is in agreement with a previous study reporting that the SDS-activatable form of PAI-1 might not be present in human platelets [33].

How, then, could the activity of PAI-1 be preserved for such a prolonged period of time in the platelet? A potential mechanism has been suggested by Lang and Schleef, who showed that platelets possess a unique mechanism for stabilization of active PAI-1, by packaging together with other large α-granule proteins in a calcium-dependent manner [12]. Active PAI-1 in plasma is stabilized by binding to vitronectin [34], [35] which has also been detected in platelet α-granules. However, some studies have failed to detect the vitronectin-PAI-1 complex in platelets [12], [17] and it is therefore controversial whether vitronectin is the stabilizing factor of PAI-1 in platelets. This issue remains to be evaluated.

From a clinical perspective, there is compelling evidence that platelet-derived PAI-1 has an important physiologic and pathophysiologic role in making platelet-rich blood clots resistant to both endogenous and pharmacological thrombolysis [5], [6], [8]. Despite this, most previous studies have reported activity levels of platelet PAI-1 that are probably far too low to explain its putative functional role. Our results may provide the missing clue to reconcile the seemingly contradictory findings. Taken together, our observations suggest that the large amount of PAI-1 stored in platelets is functional and thus capable to inhibit fibrinolysis, which may explain their observed role in clot stabilization. The present findings suggest that pre-analytic preparatory procedures have contributed to the underestimation of platelet PAI-1 activity in previous studies.

## Materials and Methods

### Subjects and Ethics Statement

Platelets were collected from healthy subjects that were not allowed to take acetyl salicylic acid or non-steroid anti-inflammatory drugs 10 days prior to the investigation. All subjects were given written and oral information and gave their verbal consent, in accordance with the study protocol that was approved (Ö498-03) by the Ethics Committee of the University of Gothenburg. All samples were handled anonymously and were not identifiable during the preparations making written consent uncalled for. Verbal consent was approved by the Ethics Committee of the University of Gothenburg.

### Platelet isolation

Platelets were isolated, washed, counted, and pelleted as described in detail previously [13]. In brief, 85 ml blood from each of 12 subjects was drawn in acid-citrate-dextrose solution containing prostaglandin E_1_ (PGE_1_). Platelet-rich plasma was prepared by repeated centrifugation at room temperature. After pelleting, the platelets were washed in Pipes-buffer containing PGE_1_. Platelets were counted, and pelleted and the supernatant was discarded. Platelets were immediately used for subsequent preparation and analysis.

### Determination of total amount of platelet PAI-1 antigen

Between 50 to 100×10^6^ platelets were lysed in lysis buffer (50 mM Tris-HCl, 50 mM NaCl, 1 mM MgCl_2_, 1 mM EDTA, 0.1% Triton® X-100 at pH 7.4) for 30 min on ice. The platelet lysates were centrifuged at 10 000×*g* for 10 minutes at 4°C to remove cell debris, and the supernatants were stored at −80°C until analysis. The total amount of PAI-1 antigen in platelet lysates was determined by Coaliza® PAI-1 ELISA (Chromogenix, Milan, Italy), according to the manufacturers' instructions. The samples were diluted with the buffers supplied as well as in PAI-1 depleted plasma (Biopool, Umeå, Sweden). Possible interference of the lysis buffer was excluded by re-analysis of the standard diluted with lysis buffer, and no interference was detected.

Two additional ELISA kits for PAI-1 antigen determinaition were also used TintElize® PAI-1 (Biopool, Umeå, Sweden), and Imubind® Plasma PAI-1 (American Diagnostica, Stamford, CT, USA).

### Functional assay of active platelet PAI-1 by Western blot analysis

Due to variations in platelet PAI-1 levels and platelet yield from each subject the amount of platelets, tPA, and the lysis volume had to be optimized in each preparation in order to obtain optimal tPA and PAI-1 concentrations for detection with Western blot. From each of the 12 donors two series of experiments were performed in which either tPA or the number of platelets were kept constant. In the average experiment, first, a total of 300×10^6^ platelets were used in ten sample preparations. The platelet pellets were resuspended in 250 µl Pipes-buffer [13] and serially increasing amounts of *sc*-tPA (Biopool, Umeå, Sweden), in steps from 25 to 300 ng, were added. In the other series of experiments, a constant amount of tPA (typically 100 ng) was used in the presence of increasing numbers of platelets (in ten steps from 25 to 600×10^6^). After addition of tPA the platelets were immediately lysed at room temperature (RT) by adding 10×lysis buffer (25 µl).

In additional experiments, the platelets were activated for 30 minutes by incubation with the PAR receptor agonist SFLLRN (24 µM) (Bachem, Bubendorf, Switzerland) both in the absence or presence of an excess of tPA.

Subsequently, the different platelet lysate or releasate samples were centrifuged at 10 000 *g* for 10 minutes at 4°C and the supernatants were diluted 1∶2 in Laemmli sample buffer with or without 35 mM SDS [36], loaded on 10% Tris-Glycine-polyacrylamide gels in Tris-glycine SDS runningbuffer and immediately separated. Proteins were blotted onto PVDF membranes (Hybond™-P Amersham Biosciences, Buckinghamshire, UK) that was subsequently incubated with either a tPA mab PAM-3 (0.3 µg/ml) or a PAI-1 mab MAI-12 (0.5 µg/ml) (Biopool, Umeå, Sweden). After incubation with a peroxidase labelled secondary antibody, bands were visualized with Super Signal® West Pico Chemiluminescent Substrate (Pierce Perbio, Rockford, IL, USA).

To determine the amount of PAI-1 in complex with tPA (*i.e.* active PAI-1) in the platelet lysate we identified the sample with the highest tPA concentration added without detection of free tPA at 68 kDa. The further increase of the tPA-PAI-1 band in the next lane was used as verification that no free tPA was present but below the detection limit of the assay in the previous lane. Serial dilutions of tPA and PAI-1 were performed to determine the detection limit of the Western blot analysis which was found to be approximately 2.5 ng for both tPA and the tPA-PAI-1 complex (data not shown).

### Functional assay of active platelet PAI-1 by ^125^I labelled tPA

Labelling of 100 µg tPA (100 µl) was performed using 500 µCi Na^125^I according to the Iodogen method [37] using Pierce® Iodination Reagent (Pierce Perbio, Rockford, IL, USA). Labeled tPA (626000 cpm/µg tPA) was separated from free Na^125^I by gel filtration on a PD-10 column and eluted in 1.25 ml Phosphate Buffered Saline (Gibco BRL, Life Technology, Paisley, UK) containing 0.01% Tween 20 (to avoid adsorption to plastic ware because of the low tPA concentration). The concentration of tPA after labelling was determined using both total protein assay (Bio-Rad, Hercules, CA, USA) and ELISA (TintElize® tPA, Biopool). Recovery of functional tPA (i.e. ability to complex PAI-1) was evaluated by addition of 10 to 10 000 fold molar excess of active rhPAI-1 (expressed in Chinese Hamster Ovary cells [38]) and labelling was shown to reduce the activity of tPA by 40%.

Platelets from 3 subjects were prepared and lysed in lysis buffer in the presence of ^125^I-labelled tPA, as described. The samples were diluted in sample buffer 1∶2 (without SDS) and separated on 10% Tris-Glycine gels. After autoradiography the tPA and tPA-PAI-1 complex were cut out and quantified by analysis on a Packard Cobra II Auto Gamma counter (Perkin-Elmer, Downers grove, Il, USA).

### Comparison of different methods for lysis of platelets

Three separate experiments were performed to evaluate the effect of different lysis methods on the platelet PAI-1 activity. Platelets were isolated as described, 180×10^6^ platelets were pelleted, re-suspended, and lysed in accordance with the previously described protocols [9], [12], [30]. Platelets were re-suspended in 1 ml homogenisation buffer [12] (150 mM NaCl, 20 mM Tris-HCl, pH 7.5, containing 2.5 mM EDTA 100 nM PGE_1_, and 5.5 mM D-glucose), 1 ml autologous plasma [9] or 1 ml Pipes-buffer with or without 500 ng tPA. The platelets were lysed either by freezing/thawing [11], [30] or by sonication [9], [10], [12]. Platelet samples were also lysed with 0.1% Triton X-100, as described. The different combinations of lysis methods and buffers are described in detail in the legends to Figure 4.

Sonication was performed using a Branson Sonifier® 250 (Branson Ultrasonic Corporation, Danbury, CT, USA) equipped with a microtip. The instrument frequency was 20 kHz and sonication was performed on ice for 5×5 s in one minute intervals with the instrument setting on either 2 or 7 (corresponding to an effect of approximately 10 and 30 W, respectively). The lysates were centrifuged at 10 000 *g* for 10 min at 4°C and the supernatants were stored at −80°C until analysis.

tPA was added to the samples lysed without tPA present and after a 15 min incubation at RT, Western blot analysis was performed as described. In two separate experiments 0.1% Triton X-100 was added to the samples after sonication or freezing/thawing to exclude the possibility that Triton X-100 interferes with the results or that incomplete lysis is the cause of low activity.

Quantitative scanning densitometry was performed using Kodak 1D Image Analysis software to estimate the relative differences in tPA-PAI-1 complex concentration in samples prepared with different lysis methods. The model net intensity of tPA-PAI-1 in sonicated or freeze/thawed samples was related to model net intensity of the tPA-PAI-1 from platelets lysed with Triton X-100.

We also performed an experiment of the effects of sonication on plasma PAI-1 activity. PAI-1 Activity Control Plasma Set from Biopool (Umeå, Sweden) was prepared according to instructions and was sonicated on ice. Samples were sonicated in duplicates 5×5 s on setting 2 or 7 in one minute intervals, or 10×5 s on setting 2 or 7 in one minute intervals. Plasma PAI-1 activity was subsequently analyzed by Chromolize™ PAI-1 activity assay (Biopool, Umeå, Sweden).

### Statistics

Results are presented as mean ± standard error of the mean.

## References

[1] Erickson LA, Ginsberg MH, Loskutoff DJ (1984). Detection and partial characterization of an inhibitor of plasminogen activator in human platelets.. J Clin Invest.

[2] Robbie LA, Bennett B, Croll AM, Brown PA, Booth NA (1996). Proteins of the fibrinolytic system in human thrombi.. Thromb Haemost.

[3] Booth NA, Robbie LA, Croll AM, Bennett B (1992). Lysis of platelet-rich thrombi: the role of PAI-1.. Ann N Y Acad Sci.

[4] Potter van Loon BJ, Rijken DC, Brommer EJ, van der Maas AP (1992). The amount of plasminogen, tissue-type plasminogen activator and plasminogen activator inhibitor type 1 in human thrombi and the relation to ex-vivo lysibility.. Thromb Haemost.

[5] Fay WP, Eitzman DT, Shapiro AD, Madison EL, Ginsburg D (1994). Platelets inhibit fibrinolysis in vitro by both plasminogen activator inhibitor-1-dependent and -independent mechanisms.. Blood.

[6] Stringer HA, van Swieten P, Heijnen HF, Sixma JJ, Pannekoek H (1994). Plasminogen activator inhibitor-1 released from activated platelets plays a key role in thrombolysis resistance. Studies with thrombi generated in the Chandler loop.. Arterioscler Thromb.

[7] Torr-Brown SR, Sobel BE (1993). Attenuation of thrombolysis by release of plasminogen activator inhibitor type-1 from platelets.. Thromb Res.

[8] Konstantinides S, Schafer K, Thinnes T, Loskutoff DJ (2001). Plasminogen activator inhibitor-1 and its cofactor vitronectin stabilize arterial thrombi after vascular injury in mice.. Circulation.

[9] Booth NA, Simpson AJ, Croll A, Bennett B, MacGregor IR (1988). Plasminogen activator inhibitor (PAI-1) in plasma and platelets.. Br J Haematol.

[10] Booth NA, Croll A, Bennett B (1990). The activity of plasminogen activator inhibitor-1 (PAI-1) of human platelet.. Fibrinolysis.

[11] Declerck PJ, Alessi MC, Verstreken M, Kruithof EK, Juhan-Vague I (1988). Measurement of plasminogen activator inhibitor 1 in biologic fluids with a murine monoclonal antibody-based enzyme-linked immunosorbent assay.. Blood.

[12] Lang IM, Schleef RR (1996). Calcium-dependent stabilization of type I plasminogen activator inhibitor within platelet alpha-granules.. J Biol Chem.

[13] Brogren H, Karlsson L, Andersson M, Wang L, Erlinge D (2004). Platelets synthesize large amounts of active plasminogen activator inhibitor 1.. Blood.

[14] Stathopulos PB, Scholz GA, Hwang YM, Rumfeldt JA, Lepock JR (2004). Sonication of proteins causes formation of aggregates that resemble amyloid.. Protein Sci.

[15] Gils A, Declerck PJ (1998). Modulation of plasminogen activator inhibitor 1 by Triton X-100–identification of two consecutive conformational transitions.. Thromb Haemost.

[16] Andreasen PA, Egelund R, Jensen S, Rodenburg KW (1999). Solvent effects on activity and conformation of plasminogen activator inhibitor-1.. Thromb Haemost.

[17] Nordenhem A, Wiman B (1997). Plasminogen activator inhibitor-1 (PAI-1) content in platelets from healthy individuals genotyped for the 4G/5G polymorphism in the PAI-1 gene.. Scand J Clin Lab Invest.

[18] Urden G, Chmielewska J, Carlsson T, Wiman B (1987). Immunological relationship between plasminogen activator inhibitors from different sources.. Thromb Haemost.

[19] Gaussem P, Grailhe P, Angles-Cano E (1993). Sodium dodecyl sulfate-induced dissociation of complexes between human tissue plasminogen activator and its specific inhibitor.. J Biol Chem.

[20] Bjorquist P, Ehnebom J, Inghardt T, Deinum J (1997). Epitopes on plasminogen activator inhibitor type-1 important for binding to tissue plasminogen activator.. Biochim Biophys Acta.

[21] Levin EG, Santell L (1987). Conversion of the active to latent plasminogen activator inhibitor from human endothelial cells.. Blood.

[22] Lindahl TL, Sigurdardottir O, Wiman B (1989). Stability of plasminogen activator inhibitor 1 (PAI-1).. Thromb Haemost.

[23] Kooistra T, Sprengers ED, van Hinsbergh VW (1986). Rapid inactivation of the plasminogen-activator inhibitor upon secretion from cultured human endothelial cells.. Biochem J.

[24] Gram J, Declerck PJ, Sidelmann J, Jespersen J, Kluft C (1993). Multicentre evaluation of commercial kit methods: plasminogen activator inhibitor activity.. Thromb Haemost.

[25] (2000). Criteria and search for specific assays for active plasminogen activator inhibitor 1 (PAI-1) in plasma.. eJIFCC.

[26] Fay WP, Murphy JG, Owen WG (1996). High concentrations of active plasminogen activator inhibitor-1 in porcine coronary artery thrombi.. Arterioscler Thromb Vasc Biol.

[27] Meijer P, Pollet DE, Wauters J, Kluft C (1994). Specificity of antigen assays of plasminogen activator inhibitor in plasma: Innotest PAI-1 immunoassay evaluated.. Clin Chem.

[28] Kluft CJ, AFH (1990). Comparison of specificieties of antigen assays for plasminogen activator inhibitor 1 (PAI-1).. Fibrinolysis.

[29] Declerck PJ, Collen D (1990). Measurement of plasminogen activator inhibitor 1 (PAI-1) in plasma with various monoclonal antibody-based enzyme-linked immunosorbent assays.. Thromb Res Suppl.

[30] Lang IM, Marsh JJ, Moser KM, Schleef RR (1992). Presence of active and latent type 1 plasminogen activator inhibitor associated with porcine platelets.. Blood.

[31] Hekman CM, Loskutoff DJ (1985). Endothelial cells produce a latent inhibitor of plasminogen activators that can be activated by denaturants.. J Biol Chem.

[32] Lambers JW, Cammenga M, Konig BW, Mertens K, Pannekoek H (1987). Activation of human endothelial cell-type plasminogen activator inhibitor (PAI-1) by negatively charged phospholipids.. J Biol Chem.

[33] Gaussem P, Angles-Cano E (1991). The formation of complexes between human plasminogen activator inhibitor-1 (PAI-1) and sodium dodecyl sulfate: possible implication in the functional properties of PAI-1.. Biochim Biophys Acta.

[34] Declerck PJ, De Mol M, Alessi MC, Baudner S, Paques EP (1988). Purification and characterization of a plasminogen activator inhibitor 1 binding protein from human plasma. Identification as a multimeric form of S protein (vitronectin).. J Biol Chem.

[35] Wiman B, Almquist A, Sigurdardottir O, Lindahl T (1988). Plasminogen activator inhibitor 1 (PAI) is bound to vitronectin in plasma.. FEBS Lett.

[36] Laemmli UK (1970). Cleavage of structural proteins during the assembly of the head of bacteriophage T4.. Nature.

[37] Fraker PJ, Speck JC (1978). Protein and cell membrane iodinations with a sparingly soluble chloroamide, 1,3,4,6-tetrachloro-3a,6a-diphrenylglycoluril.. Biochem Biophys Res Commun.

[38] Stromqvist M, Andersson JO, Bostrom S, Deinum J, Ehnebom J (1994). Separation of active and inactive forms of recombinant human plasminogen activator inhibitor type 1 (PAI-1) expressed in Chinese hamster ovary cells: comparison with native human PAI-1.. Protein Expr Purif.

